# Genome of Russian wheat aphid an economically important cereal aphid

**DOI:** 10.1186/s40793-017-0307-6

**Published:** 2017-12-28

**Authors:** Nicolaas Francois Visser Burger, Anna-Maria Botha

**Affiliations:** University of Stellenbosch, Private Bag X1, Matieland, Stellenbosch, Western Cape 7602 South Africa

**Keywords:** *Diuraphis noxia*, South Africa, Biotype comparison, Genome assembly, Arthropod genomics, SNP calling

## Abstract

Although the hemipterans (Aphididae) are comprised of roughly 50,000 extant insect species, only four have sequenced genomes that are publically available, namely *Acyrthosiphon pisum* (pea aphid), *Rhodnius prolixus* (Kissing bug), *Myzus persicae* (Green peach aphid) and *Diuraphis noxia* (Russian wheat aphid). As a significant proportion of agricultural pests are phloem feeding aphids, it is crucial for sustained global food security that a greater understanding of the genomic and molecular functioning of this family be elucidated. Recently, the genome of US *D. noxia* biotype US2 was sequenced but its assembly only incorporated ~ 32% of produced reads and contained a surprisingly low gene count when compared to that of the model/first sequenced aphid, *A. pisum*. To this end, we present here the genomes of two South African *Diuraphis noxia* (Kurdjumov, Hemiptera: Aphididae) biotypes (SA1 and SAM), obtained after sequencing the genomes of the only two *D. noxia* biotypes with documented linked genealogy. To better understand overall targets and patterns of heterozygosity, we also sequenced a pooled sample of 9 geographically separated *D. noxia* populations (MixIX). We assembled a 399 Mb reference genome (PRJNA297165, representing 64% of the projected genome size 623 Mb) using ± 28 Gb of 101 bp paired-end HiSeq2000 reads from the *D. noxia* biotype SAM, whilst ± 13 Gb 101 bp paired-end HiSeq2000 reads from the *D. noxia* biotype SA1 were generated to facilitate genomic comparisons between the two biotypes. Sequencing the MixIX sample yielded ±26 Gb 50 bp paired-end SOLiD reads which facilitated SNP detection when compared to the *D. noxia* biotype SAM assembly. Ab initio gene calling produced a total of 31,885 protein coding genes from the assembled contigs spanning ~ 399 Mb (GCA_001465515.1).

**Electronic supplementary material:**

The online version of this article (10.1186/s40793-017-0307-6) contains supplementary material, which is available to authorized users.

## Introduction

*Diuraphis noxia* (Kurdjumov), commonly known as the Russian wheat aphid, is an economically important hemipteran pest species (Hemiptera: Aphididae) afflicting wheat and barley yield in dry-land production regions [1]. *Diuraphis noxia* was first reported as a pest of small grains in South Africa during 1978 [2]. In 1986, *D. noxia* was detected in the US Texas Panhandle [3], where after it spread to 16 other states and two Canadian provinces within a few years. In 1988, *D. noxia* was recorded in Chile, by 1992 in Argentina [4] and finally spread to Australia in 2016 [5].The feeding of *D. noxia* results in foliar damage which include distinct white, yellow, purple or reddish-purple longitudinal streaks (chlorotic streaking), with severe leaf rolling in fully expanded leaves and the inhibition of leaf unfolding of developing leaves. This inability of the leaves to unfold traps the developing spike of the plant (termed “head-trapping”) which results in no seeds being produced [6, 7]. The rolling of leaves has the added unwanted effect of protecting the aphid from harsh environmental conditions (such as insecticide spraying or extreme temperatures) and from natural predators [8]. Overall, *D. noxia* infested wheat also suffers from stunted growth leading to a lowered biomass and a decrease in the number of tillers produced [6] thereby greatly affecting yield potential. Seed obtained from *D. noxia* infested wheat also tend to have lowered protein content and other negative attributes for the flour industry [9] which only adds to the economic injury of this pest. In *D. noxia*, it is common for mothers to carry both their daughters and granddaughters, as parthenogenetically produced granddaughter embryos develop directly within daughters, even before their own birth. This process allows for short *D. noxia* generation times and rapid population growth in favorable environments [1], but is thought to limit the available diversity possible within *D. noxia* populations [10]. Since its appearance in South Africa in the late 1970’s, *D. noxia* has undergone several biotypification events as there are currently five different biotypes recognized in South Africa [11, 12] and eight in the USA [13]. Biotypification, as referenced here, is when an aphid population is able to overcome previously established resistance within wheat [14]. Recently, the genome of the United States *Diuraphis noxia* biotype US2 was released [15] with an assembly size of ~395 Mb (296 Mb represented by contigs) and containing 19,097 genes. While the study was able to produce a total of 1.3 Gb of sequence data, it could only incorporate ~32% of this into an assembly comprising ~ 70% of their predicted genome size. A partial assembly due to an under estimation of genome size may explain why their values differ so greatly to that of the closest relative of *D. noxia*, *A. pisum* (37, 865 genes and 541 Mb assembly).

Here we present the genomes of the most virulent [11] South African *D. noxia* biotype SAM and its progenitor, the least virulent South African *D. noxia* biotype, SA1 [16], as well as information on the heterozygosity within geographically separated *D. noxia* populations. This study forms part of a larger survey encompassing global *D. noxia* genomic variation.

## Organism information

### Classification and features

**Fig. 1:**
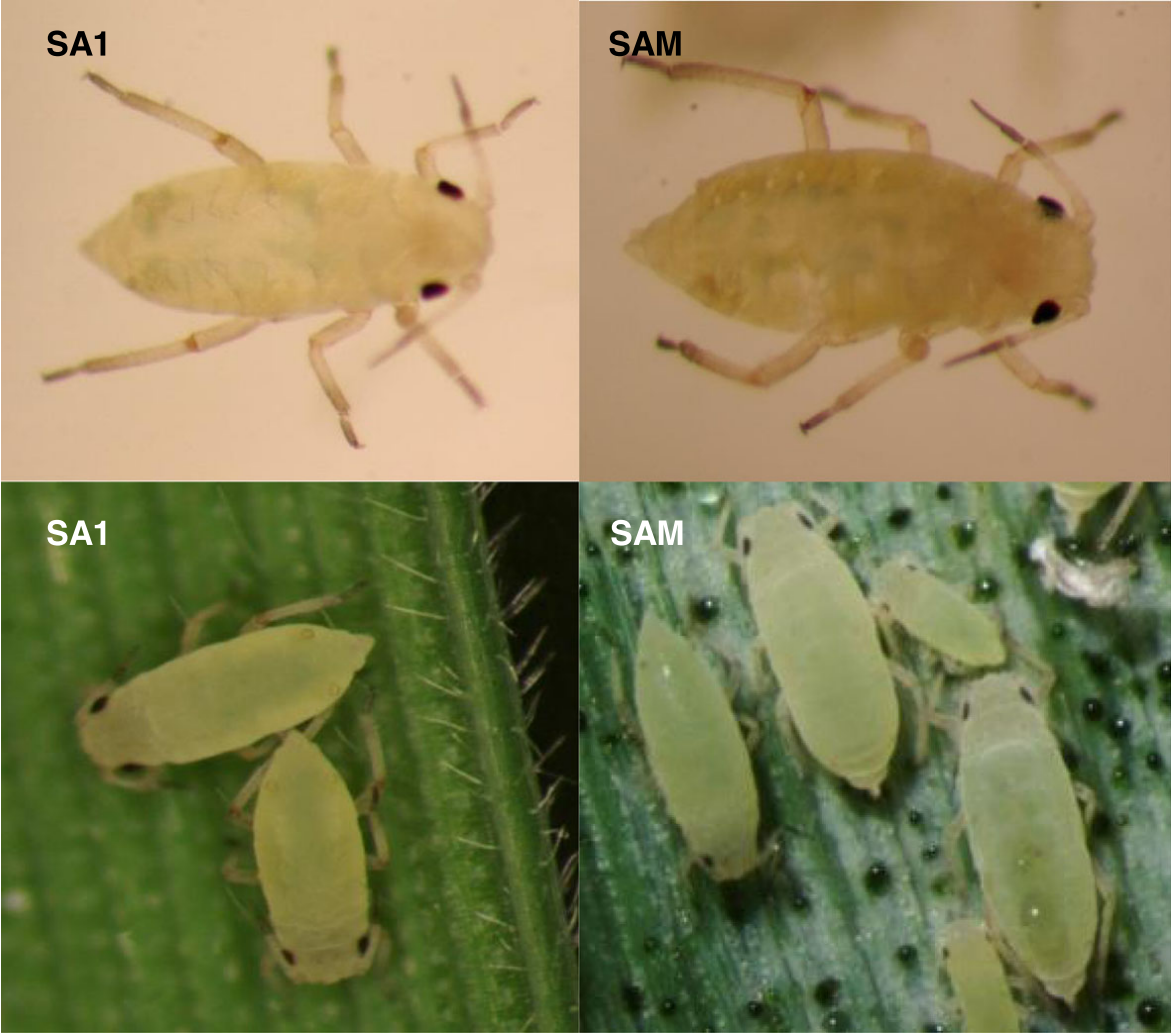
Photomicrograph of South African *Diuraphis noxia* biotypes SA1 and SAM

**Fig. 2:**
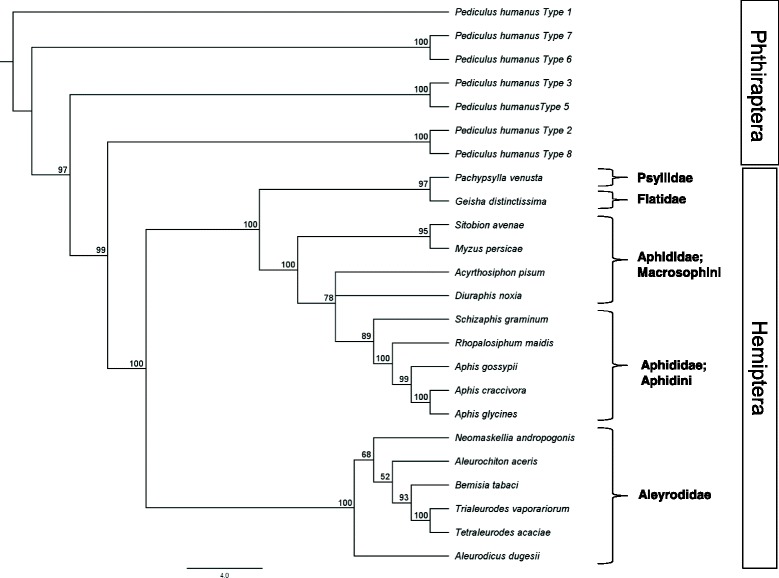
PAUP generated phylogenetic tree based on whole mitochondrial genomes. A maximum parsimony tree generated through PAUP [56] utilizing whole mitochondrial genomes, that was aligned with MAFFT [57], illustrating *Diuraphis noxia*’s close association with *Acyrthosiphon pisum*

*Diuraphis noxia* Kurdjumov (Hemiptera: Aphididae) (Table 1) is a phloem feeding Hemipteran that predominantly feeds on winter wheat and spring barley [17], with the ability to utilize other grasses as alternate hosts [3, 16]. It is pale green and up to 2 mm long with short and rounded cornicles (Fig. 1). Cornicles are structures limited to aphids on the posterior abdomen and its presence is used to assist in the identification of *D. noxia* [18]. The cornicles above the cauda give the aphid the appearance of having two tails and it is believed that these structures help aphids with predator defense [3]. Alignments using whole mitochondrial genomes [19] indicate that the closest relative of *D. noxia* is *Acyrthosiphon pisum* (Fig. 2). Reproduction of *D. noxia* can either be holocyclic (sexually reproducing males and females), as in areas where *D. noxia* is deemed endemic such as Hungary and Russia [20, 21], or anholocyclic (parthenogenic females), where *D. noxia* is deemed invasive [8]. Reproduction through asexual means can lead to a fecundity rate of between 3 and 5 aphids per day with an average lifespan of roughly 50 days, of which 9 are spent as nymphs [20]. Both forms of reproduction can lead to two morphological morphs, namely alatae (wingless forms) and apterae (winged form), with the latter form responsible for the wider geographical dispersal of the aphid [6].

**Table 1:** Classification and general features of *Diuraphis noxia* biotype SAM [22]

MIGS ID	Property	Term	Evidence code^a^
	Classification	Domain: *Metazoa*	TAS [50]
		Phylum: *Arthropoda*	TAS [51]
		Class: *Insecta*	TAS [52]
		Order: *Hemiptera*	TAS [53]
		Family: *Aphididae*	TAS [54]
		Genus: *Diuraphis*	TAS [55]
		Species: *noxia*	TAS [18]
		(Type) strain: *South African Mutant (SAM)*	TAS [11]
	Gram stain	N/A	
	Cell shape	N/A	
	Motility	N/A	
	Sporulation	N/A	
	Temperature range	N/A	
	Optimum temperature	N/A	
	pH range; Optimum	N/A	
	Carbon source	N/A	
MIGS-6	Habitat	N/A	
MIGS-6.3	Salinity	N/A	
MIGS-22	Oxygen requirement	N/A	
MIGS-15	Biotic relationship	N/A	
MIGS-14	Pathogenicity	N/A	
MIGS-4	Geographic location	South Africa	TAS []
MIGS-5	Sample collection	June 2012	NAS []
MIGS-4.1	Latitude	N/A	
MIGS-4.2	Longitude	N/A	
MIGS-4.4	Altitude	N/A	

^a^Evidence codes - IDA: Inferred from Direct Assay; TAS: Traceable Author Statement (i.e., a direct report exists in the literature); NAS: Non-traceable Author Statement (i.e., not directly observed for the living, isolated sample, but based on a generally accepted property for the species, or anecdotal evidence). These evidence codes are from the Gene Ontology project [31]

## Genome sequencing information

### Genome project history

The genome of the most virulent South African *D. noxia* biotype, SAM, was sequenced, along with that of its less virulent progenitor, biotype SA1, in an attempt to determine the genomic factors responsible for biotypification. With this, a pooled sample comprising of geographically separated *D. noxia* populations (MixIX) was also sequenced to ascertain the scope of heterogeneity experienced by the species as a whole. The draft genome sequence, as well as that all produced sequences, has been deposited at the NCBI in the GenBank database under ID GCA_001465515.1 and BioProject PRJNA297165. The project information and its association with MIGS version 2.0 compliance are summarized in Table 2 [22].

**Table 2:** Project information

MIGS ID	Property	Term
MIGS 31	Finishing quality	Level 2: High-Quality Draft
MIGS-28	Libraries used	Illumina paired-end library
MIGS 29	Sequencing platforms	Illumina HiSeq
MIGS 31.2	Fold coverage	×45 (SAM); ×22 (SA1); ×27 (MixIX)
MIGS 30	Assemblers	Soapdenovo
MIGS 32	Gene calling method	Augustus
	Locus Tag	N/A
	Genbank ID	GCA_001465515.1
	GenBank Date of Release	14/12/2015
	GOLD ID	Gp0149495
	BIOPROJECT	PRJNA297165
MIGS 13	Source Material Identifier	N/A
	Project relevance	Academic and Agricultural

### Growth conditions and genomic DNA preparation

Insectary reared strains of *D. noxia*, kept at ± 22 °C and natural lighting, were utilized for all confirmed *D. noxia* populations’ genomic DNA extractions. Genomic DNA from adult aphids of the South African *D. noxia* biotypes SAM [11] and SA1 [16], and that of the pooled MixIX sample, was used for next generation sequencing (NGS) during the study. The genomic DNA extraction of aphid DNA was conducted as follows; whole aphids were flash frozen in liquid nitrogen, ground and DNA extracted using the Qiagen DNeasy Blood and Tissue kit according to the manufacturer’s protocol [19]. The MixIX sample consisted of 2 μg of genomic DNA for each *D. noxia* representative, which consisted of three field collected South African *D. noxia* populations (SA1 < SA2 < SA3 in order of increasing virulence); one field collected Czech *D. noxia* population; two insectary reared US *D. noxia* populations (US1 < US2 in order of increasing virulence); one field collected Syrian *D. noxia* population; and two field collected Argentinian *D. noxia* populations. The integrity of the extracted DNA was then verified through electrophoresis, making use of a 1.5% agarose gel, and quantified using a Qubit v2.0 fluorometer.

### Genome sequencing and assembly

269,657,598 (biotype SAM) and 119,235,662 (biotype SA1) 101 bp reads were obtained from single paired-end libraries constructed with the Illumina TruSeq Nano DNA Library Preparation Kit, with an average 500 bp insert size, that were sequenced on the Illumina HiSeq2000 sequencing platform by Macrogen, Inc. (Seoul, Korea). Whole genomic DNA obtained from the MixIX sample produced 334,866,714 50 bp reads using the SOLiD sequencing platform from a 3–4 Kbp long mate-paired library by SEQOMICS Biotechnológia Kft. (Budapest, Hungary).

Raw sequences obtained from the Illumina HiSeq2000 sequencing of the *D. noxia* SAM biotype, and from the SOLiD system for the MixIX sample, were trimmed and filtered so that all bases had a minimum Phred score of 20. Reads mapping to 10.1601/nm.3113 of *D. noxia* [CP013259.1] and that of the mitochondrion of *D. noxia* [19] were removed from further analysis. Optimal *k*-mer length for the *D. noxia* biotype SAM assembly was determined using KMERGENIE [23], while using DSK [24] to estimate the optimal *k*-mer frequency cut-off. GCE [25] was utilized to estimate the genome size of *D. noxia* through using the optimal *k*-mer size generated by KMERGENIE and the frequency of the optimal *k*-mer size as determined by DSK. The *D. noxia* genome of biotype SAM was assembled using the SOAP de novo software package [26]. After contig assembly, scaffolds were constructed by realignment of useable paired-end reads onto the contig sequences.

**Fig. 3:**
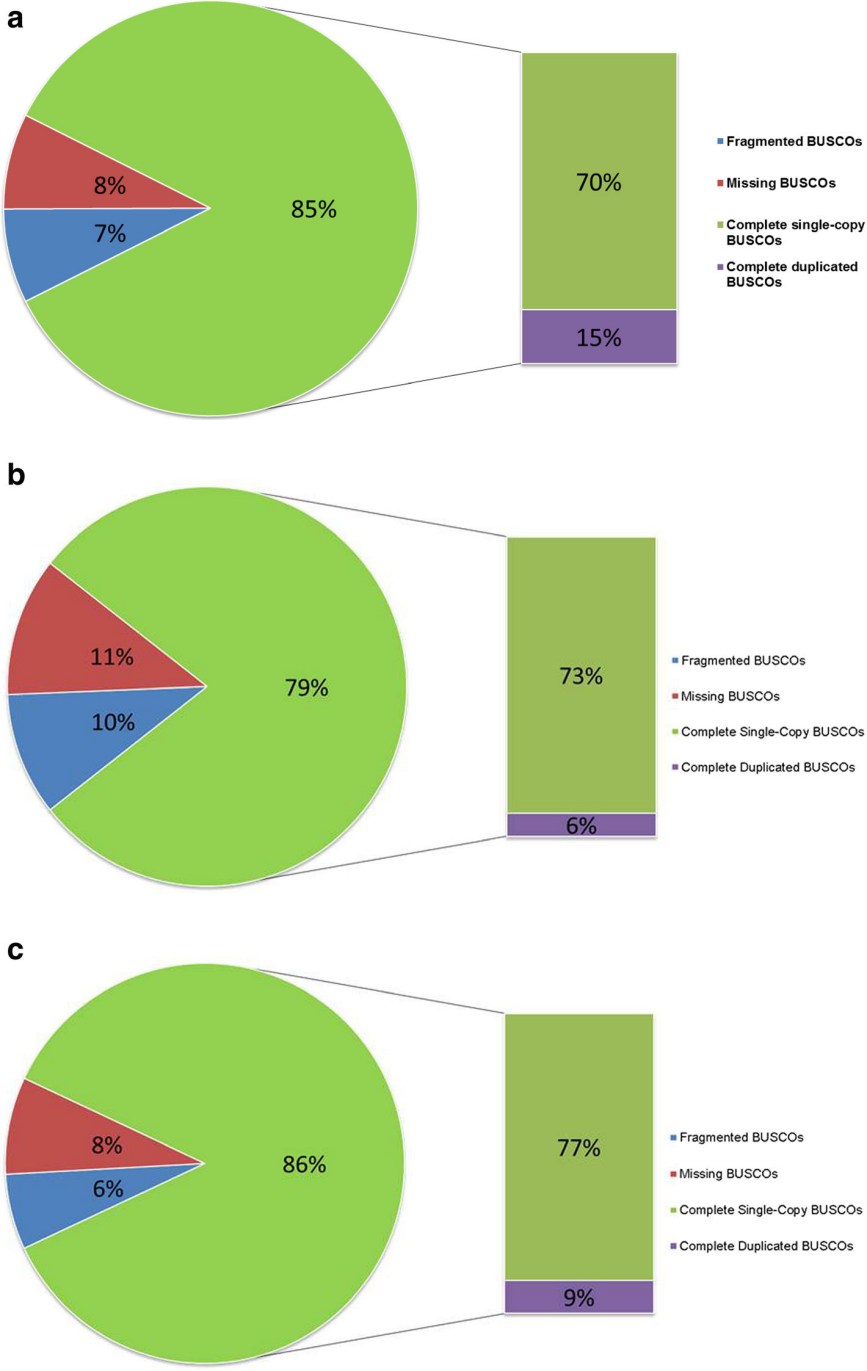
Quantitative assessment of genome assembly through BUSCO. **a** BUSCO analysis utilizing *D. noxia* biotype SAM contigs; **b** BUSCO analysis utilizing *D. noxia* biotype RWA2 scaffolds (GCA_001186385.1) [15]; and **c** BUSCO analysis utilizing *Acyrthosiphon.pisum* scaffolds (GCA_000142985.2)

Trimmed *D. noxia* biotype SAM reads were also iteratively mapped (3 iterations), using the Geneious (v7.1.5) software package [27], against the assembled scaffolds of *Acyrthosiphon pisum* (Acyr2.0) obtained from ENSEMBL [28]. The consensus sequences obtained from the reference mapping were then compared through the use of the BLASTn [29] application to the de novo contigs. Any sequences that produced no match through use of BLASTn were added to the contigs obtained from the SAM de novo assembly to build the final draft genome. A total of 190,686 contigs greater than 300 bp in length was produced with an average coverage of 44.8×, representing ~83% of the total reads generated. Using the assembled contigs, BUSCO v1.1 [30] was utilized to assess the completeness of the assembly and found that of the 2675 single-copy orthologues 85% were present, 7% were fragmented and 8% were missing (Fig. 3a). To allow for comparison, analysis using BUSCO was also performed with the scaffolds of the *D. noxia* biotype RWA2 genome (GCA_001186385.1) [15] and that of *Acyrthosiphon pisum* (GCA_000142985.2) (Fig. 3b and c respectively).

### Genome annotation

Gene prediction was performed using the ab initio gene caller Augustus [31] using the 36,195 protein coding genes of *A. pisum* (build v2.1) obtained from ENSEMBL [28] as a training set. Predicted protein coding genes were then assigned putative identity through the use of the BLASTp and BLASTx applications of the NCBI [29]. Protein coding genes were considered shared if they presented with at least 70% sequence identity over at least 70% of the total protein length. Blast2GO [32] was used to obtain the putative Gene Ontology (GO) [33] of the *D. noxia* protein coding genes predicted by Augustus. KOG [34] functional categories were assigned to predicted protein coding genes through use of the NCBI’s RPS-BLAST [35] and Conserved Domain KOG database [34], with an E-value smaller than 10e-3 accepted as significant. Protein coding genes were analyzed for their amino acid content through use of the Geneious (v7.1.5) platform [27] and CRISPR sites were predicted using the CRISPR Recognition Tool v1.1 [36].

Reads obtained from the SOLiD system were then mapped to the predicted protein coding genes of the SAM assembly to facilitate nucleotide variant calling using the Geneious (v7.1.5) software package [27]. The minimal criteria for assigning a single nucleotide polymorphism (SNP) required that the area in question had a mapping coverage of more than ×10, the variant was present in at least 2 sequences, and that the *p*-value predicted for the SNP should be smaller than 1 × 10^−6^ (calculated by first averaging the base quality of each base equal to the proposed SNP and averaging the qualities of each base not equal to the proposed SNP).

An EDTA-mediated exudation protocol [37] was used to collect phloem from uninfested, susceptible *Triticum aestivum* subsp. *aestivum* L cultivar Gamtoos-S leaves in triplicate. The exudates were blown to dryness under nitrogen at 55 °C and the residues were reconstituted in 200 ul 1 M (pH 8.0) borate buffer containing internal amino acid standards by the Central Analytical Facilities (CAF), Stellenbosch University. Ten microlitres of each reconstituted sample was derivatized using the Waters AQC derivatization kit. Derivatized amino acids were then separated and detected using a Waters Acquity UPLC fitted with an UltraTag C18 column and a photodiode array detector. Peaks were detected and integrated by the MassLynx software (Waters Corporation).

## Genome properties

The genome of female *D. noxia* consists of 5 holocentric chromosome pairs (4 autosomes and 1 sex or X chromosome) giving it an XX/XO sex determination system [38]. The final assembly totaled 399,704,836 bp which represents ~64% of the predicted genome size of between 593 and 623 Mb obtained through using GCE [25], the optimal *k*-mer size (KMERGENIE [23]) and distribution graphs (DSK [24]) (Table 3). The assembly GC content was 29.5% and ab initio gene calling, through Augustus [31], identified 31,885 protein coding genes greater than 32 amino acids in length. The total gene complement represented 66,633,929 bp of the assembly (16.67%) of which 20,316,122 (5.08%) consisted of coding domain sequence. Amino acid usage in the protein coding genes complement of *D. noxia* (Table 4) indicated that leucine followed by serine are the most frequently used amino acids, while tryptophan was the least frequently occurring amino acid. Of the 31,885 protein coding genes, 27,386 (~ 86%) sequences were putatively identified through BLASTx and BLASTp and only 12,791 (~ 47%) of these had a GO term assigned to them through Blast2GO [32].

**Table 3:** Genome statistics

Attribute	Value	% of Total
Genome size (bp)	399,704,836	64.06
DNA coding (bp)	66,633,929	16.67
DNA G + C (bp)	123,520,793	29.5
DNA scaffolds	190,686	64.06
Total genes	31,885	100
Protein coding genes	31,885	100
RNA genes	–	–
Pseudo genes	–	–
Genes in internal clusters	–	–
Genes with function prediction	12,791	40.12
Genes assigned to COGs	13,523	42.41
Genes with Pfam domains	13,877	43.52
Genes with signal peptides	1399	4.39
Genes with transmembrane helices	2957	9.27
CRISPR repeats	3	–

**Table 4:** Number of genes associated with general KOG functional categories

Code	Value	%age	Description
J	1272	3.99	Translation, ribosomal structure and biogenesis
A	1258	3.95	RNA processing and modification
K	2193	6.88	Transcription
L	1467	4.60	Replication, recombination and repair
B	729	2.29	Chromatin structure and dynamics
D	1503	4.71	Cell cycle control, cell division, chromosome partitioning
V	270	0.85	Defense mechanisms
T	3531	11.07	Signal transduction mechanisms
M	294	0.92	Cell wall/membrane biogenesis
N	55	0.17	Cell motility
U	1772	5.56	Intracellular trafficking and secretion
O	2101	6.59	Posttranslational modification, protein turnover, chaperones
C	498	1.56	Energy production and conversion
G	957	3.00	Carbohydrate transport and metabolism
E	872	2.73	Amino acid transport and metabolism
F	350	1.10	Nucleotide transport and metabolism
H	177	0.56	Coenzyme transport and metabolism
I	1232	3.86	Lipid transport and metabolism
P	734	2.30	Inorganic ion transport and metabolism
Q	377	1.18	Secondary metabolites biosynthesis, transport and catabolism
R	3740	11.73	General function prediction only
S	1528	4.79	Function unknown
–	18,362	57.59	Not in KOGs

The total is based on the total number of protein coding genes in the genome

## Insights from the genome sequence

With an AT content of 70.5%, *D. noxia* is the most AT-rich insect genome sequenced to date. This is very similar to its closest aphid relative, *A. pisum*, which has an AT content of 70.4%. A cursory comparison of the genic complement between *D. noxia* biotypes SAM and SA1 shows no differences, with SA1 reads mapping to all predicted protein coding genes, and no indication of genomic rearrangements. Genome size estimations, utilizing GCE and *k*-mer counting, were also inconclusive with both biotypes predicted to have roughly equal genome sizes. The predicted genome size of roughly 623 Mb containing 31,885 protein coding genes is also comparable to that of *A. pisum* which currently has an assembly size of 542 Mb with 36,195 protein coding genes assigned to it.

In order to assess whether there is a bias for selected amino acids during transcription in the *D. noxia* genome, we analyzed the frequency of specific amino acids and codon usage (Table 4). From the data it was evident that leucine followed by serine are the most frequently used amino acids in the predicted protein coding genes, while tryptophan was the least frequently occurring amino acid.

With regards to codon usage, leucine codons were used in the following order from most used TTA > TTG > CTT > CTG > CTA > CTC, while in the case of serine they were as follows TCA > TCT > AGT > TCG > AGC > TCC. Codons with low usage include tryptophan (TGG), cysteine (TGT > TGC) and histidine (CAT > CAC). The start codon (methionine, ATG) and stop codons (TAA, TGA and TAG) also occurred as expected at lower frequencies.

**Fig. 4:**
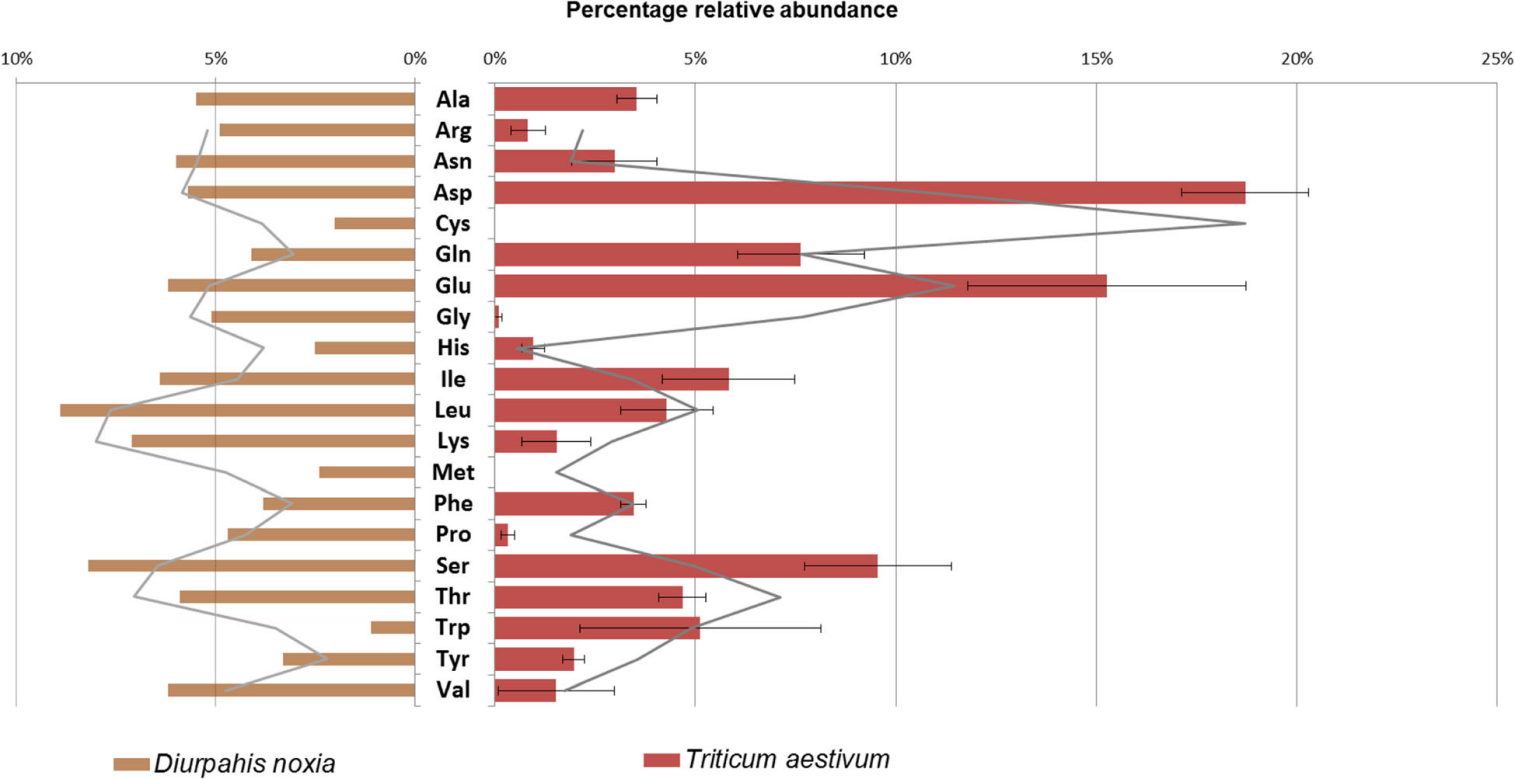
Amino acid content comparison of *Diuraphis noxia* proteins and wheat phloem. Bar plots indicate relative abundance of amino acids in *Triticum aestivum* subsp. *aestivum* L (*red*) and as component of protein coding genes within *Diuraphis noxia* (*brown*) along with two-point moving average lines

When comparing the amino acid usage of *D. noxia* protein coding genes to that of the free amino acid composition of wheat phloem (Fig. 4), it was interesting to note that of the ten most abundant amino acids present in *D. noxia* protein coding genes (in order: Leu > Ser > Lys > Ile > Glu > Val > Asn > Thr > Asp > Ala), seven were also most abundant in wheat phloem (i.e., Asp, Glu, Ser, Ile, Thr, Leu and Ala). Previous studies that either utilized an EDTA mediated phloem exudation method and/or stylectomy to investigate wheat phloem reported similar levels in unchallenged wheat plants (Additional file 1: Figure S1) [37, 39]. The apparent organization of *D. noxia* protein coding genes around the availability of free amino acids within its diet could illustrate the adaptation of the aphid towards its limited host range.

**Fig. 5:**
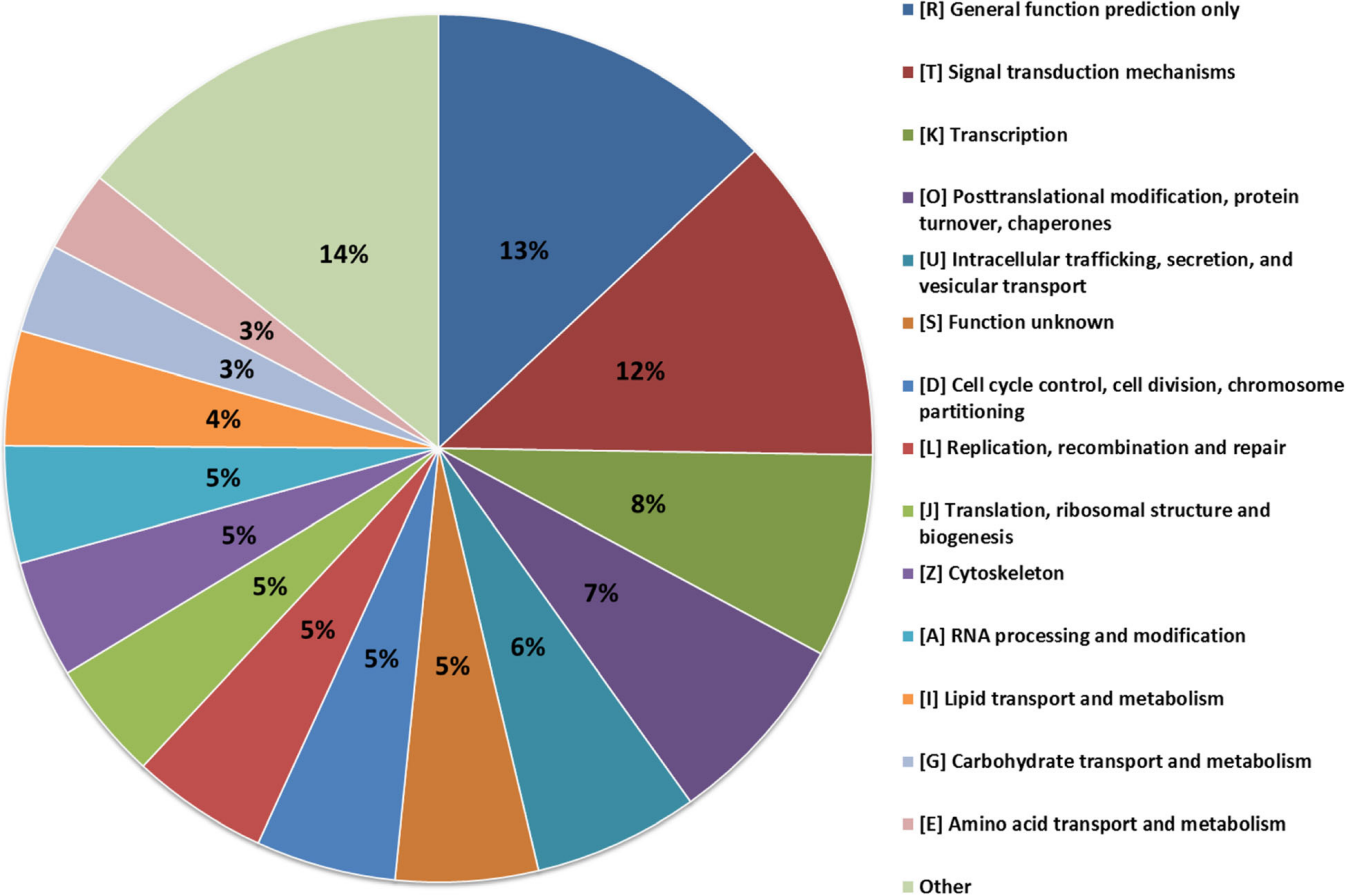
Relative abundance of KOG functional annotations within predicted genes

Assigning predicted *D. noxia* protein coding genes into KOG categories revealed that, out of the 31,885 predicted genes, 13,523 (42.42%) were successfully assigned of which the largest comprised of the general function category R (12.98%) > the signal transduction mechanisms category T (12.26%) > the transcription category K (7.61%) > posttranslational modification, protein turnover and chaperones category O (7.29%) > the intracellular trafficking, secretion, and vesicular transport category U (6.15%) (Table 5; Fig. 5). The large grouping of genes associated with protein modification and turnover is interesting in that it has been shown previously that phloem feeding aphids, despite low levels of heterogeneity, display various levels of virulence towards single host cultivars [40–43], as is the case for *Diuraphis noxia* biotypes SA1 and SAM [11, 44]. The basis for this observed variance may include the adaptability of the aphid’s salivary cohort in response to its feeding environment [45, 46] as this is central to the molecular interaction between aphids and their hosts. In a study by Lapitan et al. [47], where fractionated aphid extracts from different *D. noxia* biotypes were injected into resistant and susceptible wheat cultivars, it was found that the *D. noxia* effector(s) modulating aphid-host interactions was proteinaceous in nature and differed between biotypes. Thus *D. noxia*, as well as other Hemipterans, would require an adaptive and responsive salivary enzyme cohort that is able to adjust for their continually changing feeding environment [48].

**Table 5:** Protein amino acid constituency and codon abundancy for *Diuraphis noxia* proteins

Amino acid	Frequency	% of total	Most frequently occurring codon	Codon % of total
Ala	371,416	5.5	GCT	35.3
Cys	136,944	2.0	TGT	67.4
Asp	385,673	5.7	GAT	65.1
Glu	420,519	6.2	GAA	77.8
Phe	258,795	3.8	TTT	67.9
Gly	342,001	5.1	GGT	36.4
His	166,317	2.5	CAT	63.0
Ile	430,599	6.4	ATT	45.5
Lys	479,017	7.1	AAA	75.3
Leu	601,144	8.9	TTA	33.8
Met	161,194	2.4	ATG	100.0
Asn	403,005	6.0	AAT	66.7
Pro	318,625	4.7	CCA	41.9
Gln	278,160	4.1	CAA	69.9
Arg	330,282	4.9	AGA	32.5
Ser	555,299	8.2	TCA	26.0
Thr	400,065	5.9	ACA	35.9
Val	416,649	6.2	GTT	33.7
Trp	73,151	1.1	TGG	100.0
Tyr	220,486	3.3	TAT	62.3

### Extended insights

**Fig. 6:**
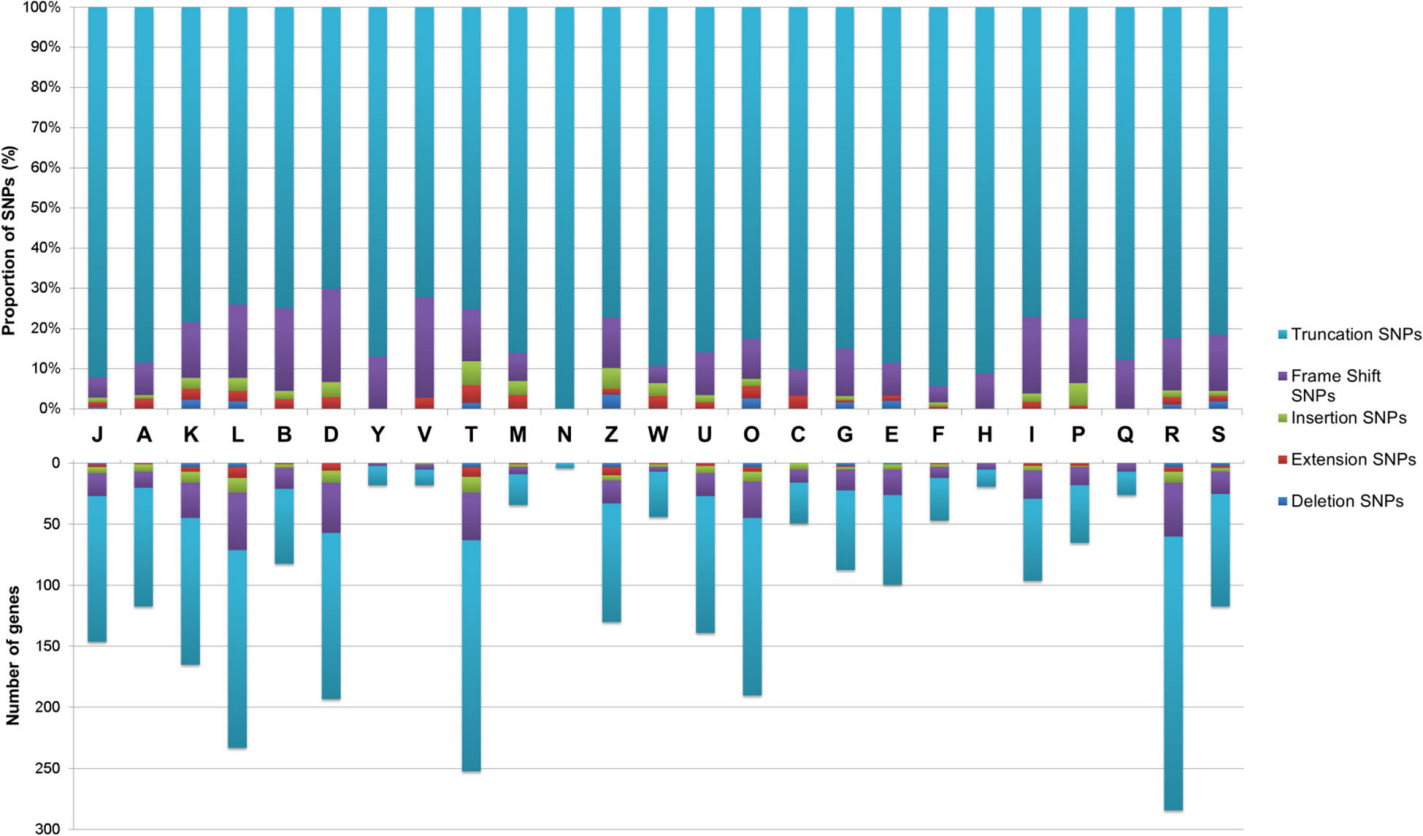
Relative abundance of SNPs within genes assigned to KOG functional categories

The pooling, and subsequent sequencing, of different *D. noxia* geographically separated populations was performed to give a clearer indication of the level of variation present overall within the species. The total number of polymorphic sites identified between the predicted protein coding genes of the South African *D. noxia* biotype SAM assembly and the MixIX sample was 92,125 (Table 6). The majority of these polymorphic sites were either synonymous (19.9%) or resulted in an amino acid substitution (68.4%). Other SNPs resulted in major underlying protein effects such as the introduction of aberrant stop codons leading to truncated transcripts (7.4%), frame shift alterations (2.6%), in-frame insertions (0.6%) and deletions (0.5%) and the extension of transcripts through disrupting stop codons (0.5%). In total, out of the predicted 31,885 protein coding genes 10,934 (34.29%) contained SNPs. The KOG general function category R (1657 genes) was assigned the most genes, followed by the translation, ribosomal structure and biogenesis category T (1434 genes); the replication, recombination and repair category L (1092); the posttranslational modification, protein turnover, chaperones category O (1061); the transcription category K (1025); and the cell cycle control, cell division, chromosome partitioning category D (938) (Fig. 6). Again, genes allocated to the general category of protein modification and turnover features prominently within the top genes containing the most SNPs, especially so when comparing SNPs with an underlying protein effect.

**Table 6:** SNPs identified between sample MixIX and *Diuraphis noxia* biotype SAM

SNP effect^a^	Value	%age of total	Number of genes	%age of genes with KOG classification
Synonymous	18,289	19.85	5677	62.37
Substitution	63,035	68.42	9674	83.54
Truncation	6844	7.43	2672	74.93
Frame shift	2375	2.58	1008	45.54
Insertion	579	0.63	163	35.58
Deletion	504	0.55	109	22.02
Extension	499	0.54	300	37.00

^a^Where synonymous SNPs cause no amino acid change, substitution SNPs cause a single amino acid substitution, truncation SNPs introduces of a stop codon, frame shit SNPs disrupt the reading frame through deletions and/or insertions of 1 or 2 bases; insertion SNPs introduces an additional codon; deletion SNPs is where a codon is removed and extension SNPs disrupt existing stop codons

The overall low number of SNPs leading to protein content variation (i.e., insertion and deletion of in-frame codons) was the least represented. This may indicate a conservation of local amino acid identity within proteins. Although SNPs resulting in an amino acid substitution were the highest recorded type of all the SNP effect types, these generally don’t incur significant functional changes. Substitutions involving amino acids possessing similar properties would constrain protein folding and target specificity. Any prediction on the underlying protein effects of these types of SNPs would also require site specific information and corroborating molecular evidence. Truncation SNPs, polymorphisms introducing aberrant stop codons within the coding domain of genes, was the third most prevalent SNP type observed (after synonymous and substitution type SNPs). Arguably, the effect of these types of SNPs can be considered more significant as they have the potential of producing transcripts of varying lengths, possibly altering the molecular action and target affinities of proteins and their underlying complexes. This could potentially afford the aphid with a wider array of “molecular machinery” to adapt to defensive responses from its host.

## Conclusions

The genome of the South African *Diuraphis noxia* biotype SAM was successfully assembled into contigs spanning roughly 400 Mb and predicted to contain 31,885 protein coding genes. A large proportion of predicted genes were assigned to KOG functional categories relating to protein modification and turnover that may help explain the differential adaptability of different *D. noxia* biotypes towards their host. The overall low variation across the genome of *D. noxia* is consistent with previous studies that have found limited variation between biotypes [48, 49]. It is though interesting that most of the functional nucleotide variation observed was predominantly present in genes governing protein modification and turnover which in turn is supportive of the adaptability of *D. noxia* when facing resistance mechanisms from its host.

## Additional file

Additional file 1: Figure S1. Comparison of amino acid profiles obtained from protein coding genes in *A. pisum* (Acyr 2.1) and the levels of amino acids in pea phloem [58]. (JPEG 59 kb)

## Abbreviations

BLAST: Basic local alignment search tool
KOG: Eukaryotic version of Clusters of Orthologous Groups (or COG)
RPS-BLAST: Reversed Position Specific BLAST

## References

[1] Morrison WP, Peairs FB (1998). Response model concept and economic impact. Response model for an introduced pest—the Russian wheat aphid.

[2] Walters MC, Penn F, du Toit F, Botha TC, Aalbersberg K, Mewitt PH, Broodryk SW. The Russian wheat aphid. Farming in South Africa. Leaflet Series. Wheat G3: 1-6. South Africa: Department of Agriculture; 1980.

[3] Stoetzel MB (1987). Information on and identification of *Diuraphis noxia* (Homoptera: Aphididae) and other aphid species colonizing leaves of wheat and barley in the United States. J Econ Entomol.

[4] Clua AR, Castro AM, Ramos SI, Gimenez DO, Vasicek AR, Chidichimo HO, Dixon AF (2004). The biological characteristics and distribution of the greenbug, *Schizaphis graminum*, and Russian wheat aphid, *Diuraphis noxia* (Hemiptera: Aphididae), in Argentina and Chile. Eur. J. Entomol..

[5] International Plant Protection Convention: Detection of Russian wheat aphid (*Diuraphis noxia*) in South Australia and Victoria. https://www.ippc.int/en/countries/australia/pestreports/2016/06/detection-of-russian-wheat-aphid-diuraphis-noxia-in-south-australia-and-victoria/. Accessed 30 Oct 2016.

[6] Burd JD, Burton RL (1992). Characterization of plant damage caused by Russian wheat aphid (Homoptera: Aphididae). J Econ Entomol.

[7] Burd JD, Burton RL, Webster JA (1993). Evaluation of Russian wheat aphid (Homoptera: Aphididae) damage on resistant and susceptible hosts with comparisons of damage ratings to quantitative plant measurements. J Econ Entomol.

[8] Botha AM (2013). A coevolutionary conundrum: the arms race between *Diuraphis noxia* (Kurdjumov) a specialist pest and its host *Triticum aestivum* (L.). Arthropod Plant Interact.

[9] Girma M, Wilde GE, Harvey TL (1993). Russian wheat aphid (Homoptera: Aphididae) affects yield and quality of wheat. J Econ Entomol.

[10] Shufran KA, Kirkman LR, Puterka GJ (2007). Absence of mitochondrial DNA sequence variation in Russian wheat aphid (Hemiptera: Aphididae) populations consistent with a single introduction into the United States. J Kans Entomol Soc.

[11] Botha AM, Burger NF, Van Eck L. Hypervirulent *Diuraphis noxia* (Hemiptera: Aphididae) biotype SAM avoids triggering defenses in its host (*Triticum aestivum*)(Poales: Poaceae) during feeding. Environ Entomol. 2014;43(3):672–81. 10.1603/EN13331 24874154

[12] Jankielsohn A. Changes in the Russian Wheat Aphid (Hemiptera: Aphididae) biotype complex in South Africa. J Econ Entomol. 2016;109(2):907-12. 10.1093/jee/tov408 26803815

[13] Burd JD, Porter DR, Puterka GJ, Haley SD, Peairs FB (2006). Biotypic variation among north American Russian wheat aphid (Homoptera: Aphididae) populations. J Econ Entomol.

[14] Smith CM, Liu X, Wang LJ, Liu X, Chen MS, Starkey S, Bai J (2010). Aphid feeding activates expression of a transcriptome of oxylipin-based defense signals in wheat involved in resistance to herbivory. J Chem Ecol.

[15] Nicholson SJ, Nickerson ML, Dean M, Song Y, Hoyt PR, Rhee H, Kim C, Puterka GJ (2015). The genome of *Diuraphis noxia*, a global aphid pest of small grains. BMC Genomics.

[16] Jankielsohn A (2011). Distribution and diversity of Russian wheat aphid (Hemiptera: Aphididae) biotypes in South Africa and Lesotho. J Econ Entomol.

[17] Brewer MJ, Elliott NC (2004). Biological control of cereal aphids in North America and mediating effects of host plant and habitat manipulations. Annu Rev Entomol.

[18] Kovalev OV, Poprawski TJ, Stekolshchikov AV, Vereshchagina AB, Gandrabur SA (1991). *Diuraphis* Aizenberg (Hom., Aphididae): key to apterous viviparous females, and review of Russian language literature on the natural history of *Diuraphis noxia* (Kurdjumov, 1913). J Appl Entomol.

[19] De Jager L, Burger NF, Botha AM (2014). Complete mitochondrial genome of *Diuraphis noxia* (Hemiptera: Aphididae) from nine populations, SNP variation between populations, and comparison with other Aphididae species. Afr Entomol.

[20] Basky Z, Jordaan J (1997). Comparison of the development and fecundity of Russian wheat aphid (Homoptera: Aphididae) in South Africa and Hungary. J Econ Entomol.

[21] Starý P, Basky Z, Tanigoshi LK, Tomanovicć Z (2003). Distribution and history of Russian wheat aphid, *Diuraphis noxia* (Kurdj.) in the Carpathian Basin (Hom., Aphididae). Anzeiger für Schädlingskunde.

[22] Field D, Garrity G, Gray T, Morrison N, Selengut J, Sterk P, Tatusova T, Thomson N, Allen MJ, Angiuoli SV, Ashburner M (2008). The minimum information about a genome sequence (MIGS) specification. Nat Biotechnol.

[23] Chikhi R, Medvedev P. Informed and automated *k*-mer size selection for genome assembly. Bioinformatics. 2013:btt310. http://kmergenie.bx.psu.edu. Accessed 18 Jul 2015. 10.1093/bioinformatics/btt310 23732276

[24] Rizk G, Lavenier D, Chikhi R. DSK: *k*-mer counting with very low memory usage. Bioinformatics. 2013:btt020. http://minia.genouest.org/dsk/. Accessed 18 Jul 2015. 10.1093/bioinformatics/btt020 23325618

[25] Binghang L, Yujian S, Jianying Y, Xuesong H, Hao Z, Nan L, Zhenyu L, Yanxiang C, Desheng M, Wei F. Estimation of genomic characteristics by analyzing *k*-mer frequency in de novo genome projects. arXiv preprint arXiv:1308.2012. 2012.

[26] Li R, Fan W, Tian G, Zhu H, He L, Cai J, Huang Q, Cai Q, Li B, Bai Y, Zhang Z (2010). The sequence and *de novo* assembly of the giant panda genome. Nature.

[27] Kearse M, Moir R, Wilson A, Stones-Havas S, Cheung M, Sturrock S, Buxton S, Cooper A, Markowitz S, Duran C, Thierer T (2012). Geneious basic: an integrated and extendable desktop software platform for the organization and analysis of sequence data. Bioinformatics.

[28] Kersey PJ, Lawson D, Birney E, Derwent PS, Haimel M, Herrero J, Keenan S, Kerhornou A, Koscielny G, Kähäri A, Kinsella RJ (2010). Ensembl genomes: extending Ensembl across the taxonomic space. Nucleic Acids Res.

[29] Altschul SF, Gish W, Miller W, Myers EW, Lipman DJ (1990). Basic local alignment search tool. J Mol Biol.

[30] Simão FA, Waterhouse RM, Ioannidis P, Kriventseva EV, Zdobnov EM. BUSCO: assessing genome assembly and annotation completeness with single-copy orthologs. Bioinformatics. 2015;31(19):3210-2. 10.1093/bioinformatics/btv351 26059717

[31] Stanke M, Morgenstern B (2005). AUGUSTUS: a web server for gene prediction in eukaryotes that allows user-defined constraints. Nucleic Acids Res.

[32] Conesa A, Götz S, García-Gómez JM, Terol J, Talón M, Robles M (2005). Blast2GO: a universal tool for annotation, visualization and analysis in functional genomics research. Bioinformatics.

[33] Ashburner M, Ball CA, Blake JA, Botstein D, Butler H, Cherry JM, Davis AP, Dolinski K, Dwight SS, Eppig JT, Harris MA (2000). Gene ontology: tool for the unification of biology. Nat Genet.

[34] Tatusov RL, Fedorova ND, Jackson JD, Jacobs AR, Kiryutin B, Koonin EV, Krylov DM, Mazumder R, Mekhedov SL, Nikolskaya AN, Rao BS (2003). The COG database: an updated version includes eukaryotes. BMC Bioinforma.

[35] Marchler-Bauer A, Lu S, Anderson JB, Chitsaz F, Derbyshire MK, DeWeese-Scott C, Fong JH, Geer LY, Geer RC, Gonzales NR, Gwadz M (2011). CDD: a conserved domain database for the functional annotation of proteins. Nucleic Acids Res.

[36] Bland C, Ramsey TL, Sabree F, Lowe M, Brown K, Kyrpides NC, Hugenholtz P (2007). CRISPR recognition tool (CRT): a tool for automatic detection of clustered regularly interspaced palindromic repeats. BMC Bioinforma.

[37] Wilkinson TL, Douglas AE (2003). Phloem amino acids and the host plant range of the polyphagous aphid, *Aphis fabae*. Entomol Exp Appl.

[38] Novotná J, Havelka J, Starý P, Koutecký P, Vítková M (2011). Karyotype analysis of the Russian wheat aphid, *Diuraphis noxia* (Kurdjumov)(Hemiptera: Aphididae) reveals a large X chromosome with rRNA and histone gene families. Genetica.

[39] Telang A, Sandström J, Dyreson E, Moran NA (1999). Feeding damage by *Diuraphis noxia* results in a nutritionally enhanced phloem diet. Entomol Exp Appl..

[40] Basky Z (2003). Biotypic and pest status differences between Hungarian and South African populations of Russian wheat aphid, *Diuraphis noxia* (Kurdjumov)(Homoptera: Aphididae). Pest Manag Sci.

[41] Zaayman D, Lapitan NL, Botha AM (2009). Dissimilar molecular defense responses are elicited in Triticum aestivum after infestation by different *Diuraphis noxia* biotypes. Physiol Plant.

[42] Lombaert E, Carletto J, Piotte C, Fauvergue X, Lecoq H, Vanlerberghe-Masutti F, Lapchin L (2009). Response of the melon aphid, *Aphis gossypii*, to host-plant resistance: evidence for high adaptive potential despite low genetic variability. Entomol Exp Appl..

[43] Lu H, Yang P, Xu Y, Luo L, Zhu J, Cui N, Kang L, Cui F (2016). Performances of survival, feeding behavior, and gene expression in aphids reveal their different fitness to host alteration. Sci Rep.

[44] Botha AM, Swanevelder ZH, Lapitan NL (2010). Transcript profiling of wheat genes expressed during feeding by two different biotypes of *Diuraphis noxia*. Environ Entomol.

[45] Miles PW (1999). Aphid saliva. Biol Rev Camb Philos Soc.

[46] Habibi J, Backus EA, Coudron TA, Brandt SL (2001). Effect of different host substrates on hemipteran salivary protein profiles. Entomol Exp Appl.

[47] Lapitan NL, Li YC, Peng J, Botha AM (2007). Fractionated extracts of Russian wheat aphid eliciting defense responses in wheat. J Econ Entomol.

[48] Puterka GJ, Black WC, Steiner WM, Burton RL (1993). Genetic variation and phylogenetic relationships among worldwide collections of the Russian wheat aphid, *Diuraphis noxia* (Mordvilko), inferred from allozyme and RAPD-PCR markers. Heredity.

[49] Swanevelder ZH, Surridge AK, Venter E, Botha AM (2010). Limited endosymbiont variation in *Diuraphis noxia* (Hemiptera: Aphididae) biotypes from the United States and South Africa. J Econ Entomol.

[50] Nielsen C, Scharff N, Eibye-Jacobsen D (1996). Cladistic analyses of the animal kingdom. Biol J Linnean Soc.

[51] Stys P, Zrzavy J (1994). Phylogeny and classification of extant Arthropoda: review of hypotheses and nomenclature. Eur J Entomol.

[52] Labandeira CC, Sepkoski JJ (1993). Insect diversity in the fossil record. Science.

[53] Dolling WR (1991). The Hemiptera.

[54] Heie O. Aphid ecology in the past and a new view on the evolution of Macrosiphini. In: Leather SR, Watt AD, Mills NJ, Walters KFA, editors. Individuals, populations and patterns in ecology. Andover: Intercept; 1994.

[55] Aizenberg, 1935. Zap. Bolshev biol. Stan. Nos. 7–8: 157. Obtained from Nomenclator Zoologicus 7:94; http://ubio.org/NZ/search.php?search=aizenberg+&quickSearch=QuickSearch&selectall=Check+All&colname=on&colcategory=on&colauthority=on&colcomments=on&page=&vol. Accessed 25 June 2017.

[56] Swofford DL (2002). PAUP*. Phylogenetic Analysis Using Parsimony (*and Other Methods). Version 4.

[57] Katoh K, Standley DM (2013). MAFFT multiple sequence alignment software version 7: improvements in performance and usability. Mol Biol Evol.

[58] Sandström J, Pettersson J (1994). Amino acid composition of phloem sap and the relation to intraspecific variation in pea aphid (*Acyrthosiphon pisum*) performance. J. Insect Physiol..

