# Supplementary figures and images for: Genome of Russian wheat aphid an economically important cereal aphid
Source: Stand Genomic Sci. 2017 Dec 28;12:90. doi: 10.1186/s40793-017-0307-6 (PMC5745598; doi:10.1186/s40793-017-0307-6)

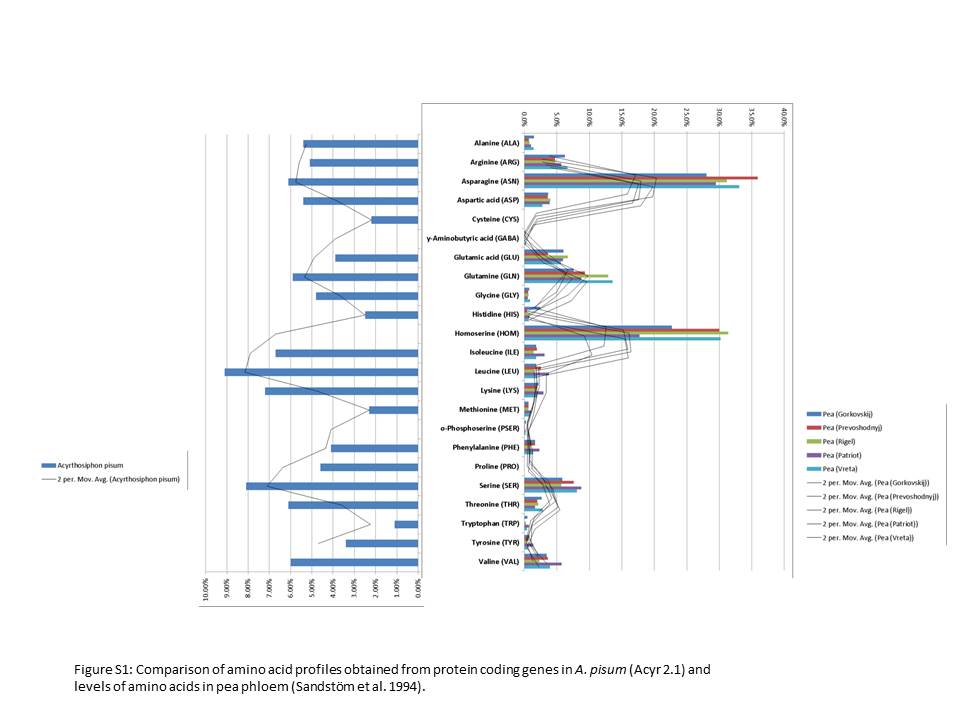

Supplement: Additional file 1: Figure S1. — Comparison of amino acid profiles obtained from protein coding genes in A. pisum (Acyr 2.1) and the levels of amino acids in pea phloem [58]. (JPEG 59 kb) [file 40793_2017_307_MOESM1_ESM.jpg]
